# SARS-CoV-2 Genomic Surveillance in Brazil: A Systematic Review with Scientometric Analysis

**DOI:** 10.3390/v14122715

**Published:** 2022-12-05

**Authors:** Diego Menezes, Paula Luize Camargos Fonseca, João Locke Ferreira de Araújo, Renan Pedra de Souza

**Affiliations:** Grupo de Pesquisa em Bioestatística e Epidemiologia Molecular, Laboratório de Biologia Integrativa, Programa de Pós-Graduação em Genética, Departamento de Genética, Ecologia e Evolução, Instituto de Ciências Biológicas, Universidade Federal de Minas Gerais, Belo Horizonte 31270-901, Brazil

**Keywords:** COVID-19, molecular epidemiology, next-generation sequencing

## Abstract

Several studies have monitored the SARS-CoV-2 variants in Brazil throughout the pandemic. Here, we systematically reviewed and conducted a scientometric analysis of the SARS-CoV-2 genomic surveillance studies using Brazilian samples. A Pubmed database search on October 2022 returned 492 articles, of which 106 were included. Ninety-six different strains were reported, with variant of concern (VOC) gamma (*n* = 35,398), VOC delta (*n* = 15,780), and the variant of interest zeta (*n* = 1983) being the most common. The top three states with the most samples in the published articles were São Paulo, Rio de Janeiro, and Minas Gerais. Whereas the first year of the pandemic presented primary circulation of B.1.1.28 and B.1.1.33 variants, consecutive replacements were observed between them and VOI zeta, VOC gamma, VOC delta, and VOC omicron. VOI mu, VOI lambda, VOC alpha, and VOC beta were also detected but failed to reach significant circulation. Co-infection, re-infection, and vaccine breakthrough reports were found. Article co-citation differed from the co-authorship structure. Despite the limitations, we expect to give an overview of Brazil’s genomic surveillance studies and contribute to future research execution.

## 1. Introduction

The coronavirus disease (COVID-19) is caused by severe acute respiratory syndrome coronavirus 2 (SARS-CoV-2) infection, and was first reported in December 2019. The SARS-CoV-2 virus had been registered in 197 countries by March 2020, when the World Health Organization declared the novel coronavirus outbreak a global pandemic. More than 450 million cases and 6.0 million deaths have been reported worldwide [1]. Although the pandemic remains of concern, SARS-CoV-2 fatalities reduction has been observed mainly due to vaccination programs. Brazil has been heavily affected by COVID-19 [2,3]. Almost 35 million cases and 700,000 deaths have been documented since the first Brazilian case [4].

Viral genetics and evolution have been the main researched topics since the first viral genome was published [5]. Sequencing studies have promptly found viral diversity. Viral nomenclature was standardised in 2020 [6]. In 2021, Greek alphabet letters were introduced [7] to enable clear communication regarding lineages that presented alarming structural mutations: variants of interest (VOIs) and concern (VOCs) [8]. An unprecedented genomic surveillance effort was carried out [9], strengthened by the development, repositioning, and availability of sequencing services (e.g., databases, analysis tools, and other bioinformatics resources) [10,11,12]. A similar trend was observed among research groups previously conducting viral genomic surveillance studies in Brazil [13,14,15]. Here, we systematically reviewed and conducted scientometric analyses using the SARS-CoV-2 genomic surveillance studies characterising Brazilian samples.

## 2. Materials and Methods

The protocol for this systematic review was registered on PROSPERO [16] (accessible at www.crd.york.ac.uk/PROSPERO/display_record.asp?ID=CRD42021273259 (accessed on 1 December 2022)). Preferred Reporting Items for Systematic reviews and Meta-analysis (PRISMA) guideline was adopted [17]. Study selection was carried out in three phases: identification, screening, and eligibility. Identification was performed on the PubMed database using a structured search argument on 5 October 2022 (Supplementary Material S1). Two independent researchers conducted the screening of the articles. A third researcher solved disagreements. Inclusion criteria were primary articles that address the frequency of SARS-CoV-2 lineages by genotyping and sequencing data using samples from any location of the Brazilian territory. In contrast, exclusion criteria were reviews and primary articles with no SARS-CoV-2 lineage detection or studies with samples from elsewhere.

Two independent researchers conducted metadata extraction. We aimed to obtain the majority of available information since there is no standardisation on reporting genomic surveillance studies. We collected data regarding the: (1) publishing process (author affiliations and State of origin), (2) description of the samples—including either genotyping data or novel genomes used in the posterior analysis in the ‘sample’ category (size, initial and final collection dates, symptomatology, travel history, and nature -human or sewage samples), and (3) genomic surveillance execution (diagnostic gene targets, sequencing platform and metrics, variant calling, and phylogeny method used). Study designs were evaluated with the Joanna Briggs Institute (JBI) Critical Appraisal Tools for Systematic Reviews Checklist for Case Reports or Studies Reporting Prevalence [18]. Statistical descriptive analysis was carried out using software R (Vienna, Austria) (version 4.1.2). The scientometric evaluation was conducted using all included manuscripts in the systematic review. Bibliographic data was downloaded through Europe PMC API using the article digital object identifier (DOI) on 19 October 2022. The association strength was the normalisation method, and clustering was performed using default values on VOSviewer (Leiden, The Netherlands) (version 1.6.17) [19]. The connection between nodes in the maps represents co-authorship (for authors and authors’ affiliation) or citation (for articles). VOSviewer employs a distance-based visualisation of similarities mapping technique to construct a map [19].

## 3. Results

### 3.1. Description of the Included Studies

**Figure 1:**
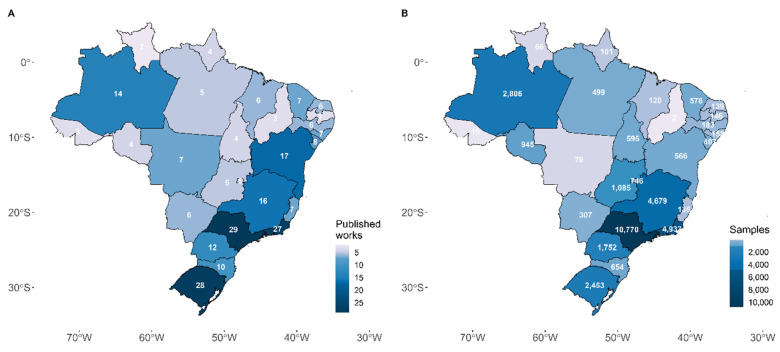
Spatial overview of the genomic surveillance in Brazil. (**A**) The number of published articles with samples from each Brazilian State. (**B**) The number of characterised samples from each Brazilian State in the published reports.

**Figure 2:**
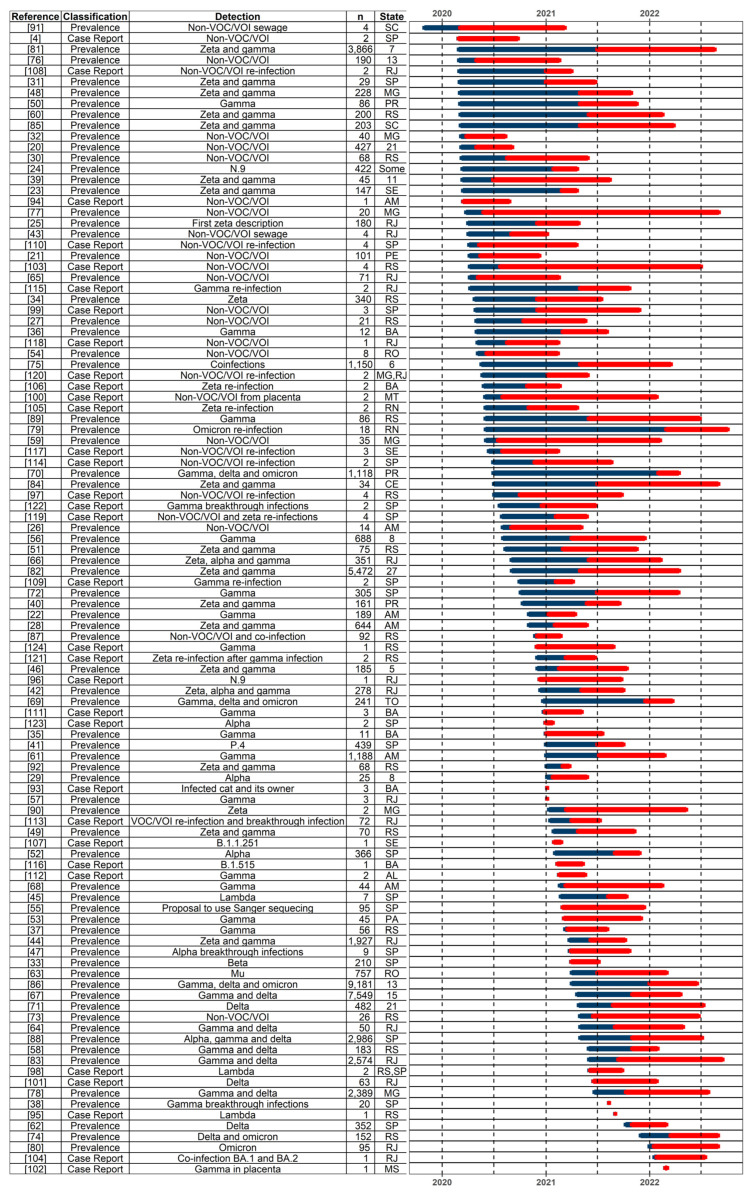
Included studies in the systematic review. Studies are ordered by the beginning of the sampling periods when available. If not, the publication date was used. Non-VOC/VOI detection was omitted when the study reported VOI or VOC. The number of sampled States is shown when there are three or more States. Blue lines represent the sampling period. Red lines represent the time between the end of sampling and the publication date. Dashed lines indicate six months intervals from 1 January 2020. AL—Alagoas, AM—Amazonas, BA—Bahia, CE—Ceará, MG—Minas Gerais, MS—Mato Grosso do Sul, MT—Mato Grosso, PA—Pará, PE—Pernambuco, PR—Paraná, RJ—Rio de Janeiro, RN—Rio Grande do Norte, RO—Rondônia, RS—Rio Grande do Sul, SC—Santa Catarina, SE—Sergipe, SP—São Paulo, TO—Tocantins, VOC—variant of concern, and VOI—variant of interest.

The search led to the screening of 492 articles (Figure S1). Our review included 106 studies, of which 73 were classified as prevalence studies [20,21,22,23,24,25,26,27,28,29,30,31,32,33,34,35,36,37,38,39,40,41,42,43,44,45,46,47,48,49,50,51,52,53,54,55,56,57,58,59,60,61,62,63,64,65,66,67,68,69,70,71,72,72,73,74,75,76,77,78,79,80,81,82,83,84,85,86,87,88,89,90,91] and 33 were case reports [4,92,93,94,95,96,97,98,99,100,101,102,103,104,105,106,107,108,109,110,111,112,113,114,115,116,117,118,119,120,121,122,123] (Table S1). The quality assessment indicated a systematic lack of participant demography and history description in case reports. At the same time, the most critical issue in the prevalence studies was the description of the participants (Figure S2). São Paulo (*n* = 29, 11.55%), Rio Grande do Sul (*n* = 28, 11.16%), and Rio de Janeiro (*n* = 27, 10.76%) were the sampled States with the most significant number of publications (Figure 1A), with Southeastern region being the most represented (*n* = 20,517, 58.77%). The top three states with the most samples included in published articles were São Paulo (*n* = 10,770; 30.85%), Rio de Janeiro (*n* = 4933; 14.13%), and Minas Gerais (*n* = 4679; 13.4%) (Figure 1B). Most studies included less than 500 samples (*n* = 92; 86.79%) (Figure S3) sampled from a single state (*n* = 91; 85.84%) (Figure 2).

In total, included studies contained 49,409 samples in the included studies (GISAID had 3.7 times more sequences uploaded), of which 571 (1.16%) were present in studies published in 2020, 7655 (15.49%) in 2021, and 41,183 (83.35%) in 2022 (Table S2). Ninety-six different strains were reported, with variant of concern (VOC) gamma (*n* = 35,398), VOC delta (*n* = 15,780), and variant of interest (VOI) zeta (*n* = 1983) being the most common thus far (Table S3). Most studies presented complete information on sample collection date ranges (*n* = 99, 94.34%), with sampling mostly starting in 2020 (*n* = 64; 60.38%) and 2021 (*n* = 33, 31.13%) (Figure 2). The median sampling period was 108.5 days, and the median time between the end of sampling and the publication of 200.5 days.

Several study features were compiled (Table S4). We have observed heterogeneity in methodological features and what authors have decided to report across studies. Clinical data were unavailable in 44 studies (41.51%), whereas outcomes of interest (e.g., death) were almost inexistent. Public laboratories (*n* = 91; 85.84%) and universities (*n* = 86, 81.13%) participated in most studies. Two studies were conducted in sewage samples. Pangolin [10] was the leading variant-calling software (*n* = 75, 70.75%), followed by IQTREE [124] (*n* = 59, 50.66%) and Nextclade [125] (*n* = 29, 27.35%). Most studies presented phylogenetic reconstructions (*n* = 84, 79.24%), with maximum-likelihood models being the most common (*n* = 79, 74.52%). Short-read platforms (e.g., Illumina MiSeq) account for more than half of the studies. The sequencing metrics were not reported in 56 studies (52.83%). The minimum coverage distribution was concentrated below 3000×, and the minimum genome span was between 75–97% (Figure S3).

### 3.2. Variant Detection in the Included Studies

The SARS-CoV-2 genomic surveillance in Brazil started in February 2020 in São Paulo, with the first reported patients returning from international destinations [4]. However, a study indicated SARS-CoV-2 mRNA sewage detection in Santa Catarina in November 2019 [90]. Communitarian transmission quickly overcame the importation rate [20,80]. Different SARS-CoV-2 strains descending from clade B.1, which present the structural mutation D614G in the Spike protein, became the most frequent variants detected in the prevalence studies during the first wave in 2020, especially B.1.1.28 and B.1.1.33 [20,21,22,23,24,25,26,27,30,31,32,34,36,39,43,48,50,51,54,56,59,60,65,66,70,72,75,76,78,80,81,83,84,88,90]. One sewage study also found B.1.1.33 circulation in Rio de Janeiro in mid-2020 [43]. The earliest reported re-infection cases were in São Paulo [109] and Rio de Janeiro [107], and co-infections were in Rio Grande do Sul [86], still in 2020.

Cases started to increase again in November 2020. Later that month, the World Health Organization designated VOIs and VOCs. VOI Zeta’s first detection was in Rio de Janeiro, with an inferred emergence date in July 2020 [25]. Zeta was found in several Brazilian States and became the most common variant until February 2021 [23,25,28,31,34,39,40,42,44,46,48,49,51,60,66,80,81,83,84,89,91], being associated with co-infections in Ceará, Rio Grande do Sul, and Bahia [75]. January 2021 was marked by a death surge that peaked in February 2021, coinciding with multiple detections of the recently described VOC gamma [22,28]. That VOC was the most common variant in the first 2021 semester throughout Brazil, associated with increased COVID-19 mortality [22,23,28,31,35,36,37,38,39,40,42,44,46,48,49,50,51,53,56,57,58,60,61,64,66,67,68,69,70,72,77,80,81,82,83,84,85,87,88,91]. In August 2021, VOC delta’s first case was confirmed in Brazil, later spreading all over Brazil [58,62,64,67,69,70,71,74,77,82,85,87]. The gamma replacement was associated with an unexpected substantial decrease in cases and deaths observed up to December 2021.

VOI mu [63], VOI lambda [45,94,97], VOC alpha [29,42,52,66,87], and VOC beta [33] were also detected in Brazil but failed to reach significant circulation. Besides variant detection and prevalence, two crucial issues were explored in the included studies during 2021: re-infections and vaccine breakthrough infections. Re-infections were described in several States as being frequently associated with VOI/VOC infection [96,104,105,108,112,113,114,116,118,119,120]. Vaccine breakthrough studies were less common, with reports found for zeta infections in Rio de Janeiro [112], gamma in Sao Paulo [38,121] and Rio de Janeiro [112], and alpha in São Paulo [47]. Later studies explored clinical outcomes in vaccinated subjects with delta infections [77].

VOC omicron’s first cases were detected at the end of November 2021, with significant circulation reported by December 2021. In 2022, the third and fourth case waves were observed, with daily reported cases reaching over 250,000 in January. Six studies characterised omicron circulation in several States [69,70,74,79,85]. Omicron BA.1/BA.2 co-infection in Rio de Janeiro [103] and BA.1 re-infection in Rio Grande do Norte [78] were reported. A study with samples from Paraná did not indicate changes in lethality during the omicron wave [70]. Further studies will likely be published to uncover further details of the omicron circulation and its sub-variants.

### 3.3. Scientometric Analysis

**Figure 3:**
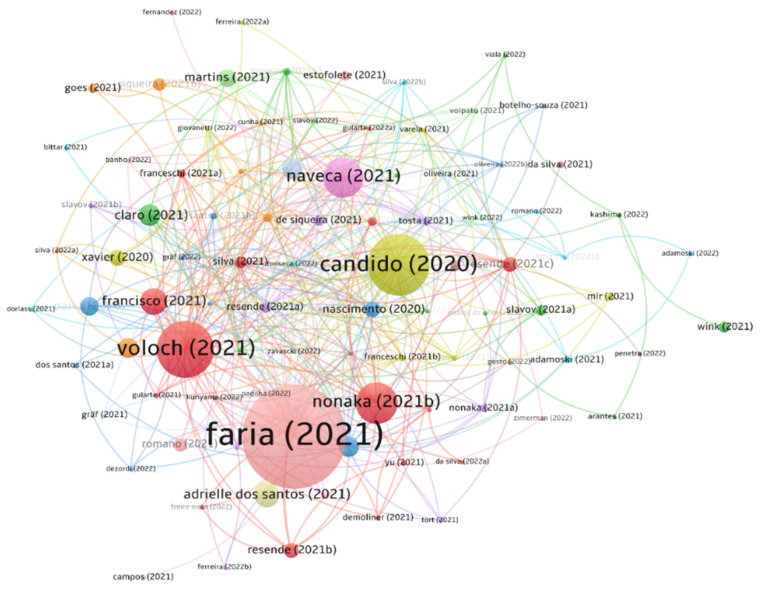
Citation map for articles cross-citations. Connectors indicate article citations. The circle size is weighted by the citation number of each study in the literature up to 25 October 2022. Thirteen clusters were found, and are indicated by different colours. Metadata is available in Table S5 [20,21,22,23,24,25,27,28,29,30,31,32,33,34,35,36,37,38,39,40,41,42,44,45,46,47,48,49,50,51,52,53,54,56,57,58,59,60,61,63,64,65,66,67,68,69,70,72,72,73,74,75,76,77,78,79,80,81,83,84,85,86,87,88,89,90,91,93,94,95,96,97,98,99,100,101,102,104,105,106,107,108,109,110,111,112,114,116,118,119,120,121,122,123].

The first scientometric analysis evaluated cross-citations among the included articles (Figure 3). We identified 13 clusters with 338 cross-citations in the network comprised of 95 articles. Eleven manuscripts did not cite another included article. The publications with the highest number of cross-citations were [22] (*n* = 49), [20] (*n* = 38), [28] (*n* = 36), and [25] (*n* = 26), whereas the highest overall number of citations in the literature until October 2022 was observed in [22] (*n* = 433), [20] (*n* = 182), [26] (*n* = 161), and [105] (*n* = 95) (Table S5).

**Figure 4:**
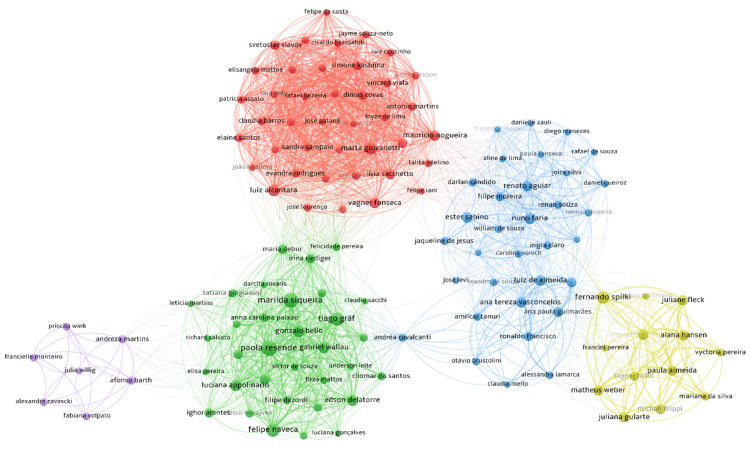
Author co-authorship map. Authors with at least four articles were included (*n* = 127 out of 1190). Connectors indicate co-authorships. Circle sizes were weighted by the number of publications included in the systematic review. Five clusters were found, and are characterised by different colours. Metadata is available in Table S6.

**Figure 5:**
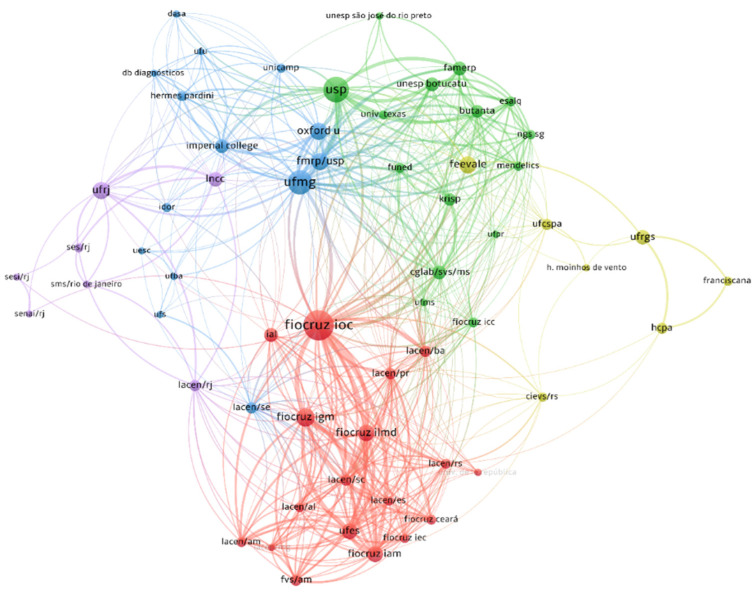
Organisation co-authorship map. Organisations with at least three articles in the systematic review were included (*n* = 63 out of 229). Connectors indicate co-authorship. The number of included articles from each organisation weighted circle sizes. Five clusters were found, and are indicated by different colours. Metadata is available in Table S7.

The second scientometric analysis showed authors who published together (Figure 4). The co-authorship network contains 127 authors in five clusters (Table S6). Five groups were also observed in the authors’ affiliation co-authorship network (Figure 5). We also created networks with keywords (Figure S4) and journals (Figure S5).

## 4. Discussion

Genomic surveillance in Brazil has been a prolific field. Here, we were able to review information from 106 publications. We know that the published literature does not fully cover all surveillance conducted, since the shared sequences in GISAID even outnumber the official governmental data in Brazil, a situation only replicated in the USA [126]. Despite the convenience of the pathogen databases, publishing articles on the field is fundamental to reporting the details of the execution process, which is the most reliable way to improve the design of future works.

We identified heterogeneity in sample representation across states. Overrepresentation in the southeast region reflects more extensive infrastructure, human resources, and previous experience executing viral genomic surveillance [13,14,15]. On the other hand, the protagonism of public institutions was homogenous, reinforcing their relevance in generating reliable data to subsidise accurate decision-making in public policy. In the future, stable funding will be required to expand the sequencing capacity and effectiveness in monitoring SARS-CoV-2 and other pathogens of medical interest.

Another interesting trend was that most studies sampled only one state. Broader studies are essential to describe epidemiological diversity under a uniform design and execution. The appropriate report in these works has the potential to reveal local challenges faced during the research process in each State from a comparative perspective. On the other hand, local studies were an essential part of the SARS-CoV-2 monitoring in the country, giving faster, deeper, and more precise responses to the COVID-19 pandemic in different regional scenarios.

Gathering study characteristics may help researchers design future studies. However, we found heterogeneity in the reported items, which made it difficult to conduct proper comparisons between studies. Genomic surveillance still lacks a specific extension of the Strengthening the Reporting of Observational Studies in Epidemiology (STROBE) statement [127]. The closest instrument would be the STROBE—Molecular Epidemiology [128], which does not include relevant information (e.g., sequencing parameters, bioinformatic analysis details).

The scientometric analysis indicated a different structure between co-authorships and article co-citations which could be interpreted as a good indicator of citations happening outside the research groups. However, we still observed articles that did not cite other Brazilian genomic surveillance studies converging in what Clarke and Chalmers once described as “islands in search of continents” [129]: a lack of connection between what is being newly described and what is known in the subject. Whenever possible, original studies could use systematic reviews to contextualise their findings [130].

Our work has limitations. The search argument, although aiming to return the most complete and, at the same time, precise search results, can be too stringent, and we may have missed relevant studies. The area is quickly evolving, and manuscripts have been published with previous variants [131,132] and XAG recombinants while we were updating this systematic review [133,134].

Although the genomic surveillance findings present biological significance, our work is significant from another perspective. Since it is hard to keep track of articles because so many have been published, authors can get a quick field overview, being able to plan new experiments, compare technical aspects, or discuss their results using the existing literature. Therefore, this review reduces initial information overload, accelerating and improving scientific research.

## Data Availability

Not applicable.

## Supplementary Materials

The following supporting information can be downloaded at: https://www.mdpi.com/article/10.3390/v14122715/s1. Figure S1: PRISMA flowchart. Figure S2: Quality assessment using the JBI Critical Appraisal Tools in Case Reports (A) and Prevalence Studies (B). Figure S3: Sequencing metrics of the studies included in this review. Figure S4: Keyword co-occurrence map. Figure S5: Journal citation map. Table S1: Dataset with the 106 selected studies. Table S2: Amount of samples in the 106 articles versus sequences included in the GISAID platform from 2020 to 2022. Table S3: Absolute frequencies of all the lineages reported in the 106 selected studies. Table S4: Descriptional panel of nine variables concerning the 106 studies included grouped by study type. Table S5: Studies included in Figure 3. Table S6: Authors included in Figure 4. Table S7: Organizations included in Figure 5. Table S8: Top 30 articles cited in the 106 articles included in the systematic review.
