# Supplementary material for: SARS-CoV-2 Genomic Surveillance in Brazil: A Systematic Review with Scientometric Analysis
Source: Viruses. 2022 Dec 5;14(12):2715. doi: 10.3390/v14122715 (PMC9784312; doi:10.3390/v14122715)

# SARS-CoV-2 genomic surveillance in Brazil: a systematic review with scientometric analysis

Diego Menezes [1]; Paula Luize Camargos Fonseca[1]; João Locke Ferreira de Araújo[1]; Renan Pedra de Souza [1]

[1] Grupo de Pesquisa em Bioestatística e Epidemiologia Molecular; Laboratório de Biologia Integrativa; Programa de Pós Graduação em Genética; Departamento de Genética, Ecologia e Evolução; Instituto de Ciências Biológicas; Universidade Federal de Minas Gerais

**Supplementary material S1**

**Search strategy**

(SARS-CoV-2[Title/Abstract] OR 2019-nCoV [Title/Abstract] OR SARS-CoV2 [Title/Abstract])

AND

("Genomic Structural Variation/genetics"[Mesh] OR "Evolutionary dynamics" [Title/Abstract] OR phylodynamics [Title/Abstract] OR phylogeny [Title/Abstract] OR "phylodynamic analysis" [Title/Abstract] OR "genomic surveillance"[Title/Abstract] OR evolution [Title/Abstract] OR "molecular epidemiology" [Title/Abstract] OR "genomic epidemiology" [Title/Abstract] OR sequencing [Title/Abstract] OR variant [Title/Abstract] OR variants [Title/Abstract] OR strains [Title/Abstract] OR lineage [Title/Abstract] OR strain [Title/Abstract] OR lineages [Title/Abstract])

AND

("Brazil"[Mesh] OR Brazil [Title/Abstract] OR Brazilian [Title/Abstract] OR Manaus [Title/Abstract] OR "São Paulo" [Title/Abstract] OR "Rio de Janeiro" [Title/Abstract] OR Amazonas [Title/Abstract] OR "Minas Gerais" [Title/Abstract] OR "Espírito Santo" [Title/Abstract] OR Pará [Title/Abstract] OR Rondônia [Title/Abstract] OR Roraima [Title/Abstract] OR Acre [Title/Abstract] OR Amapá [Title/Abstract] OR Tocantins [Title/Abstract] OR "Rio Grande do Norte" [Title/Abstract] OR Ceará [Title/Abstract] OR Sergipe [Title/Abstract] OR Alagoas [Title/Abstract] OR Pernambuco [Title/Abstract] OR Bahia [Title/Abstract] OR Paraíba [Title/Abstract] OR Piauí [Title/Abstract] OR Maranhão [Title/Abstract] OR "Mato Grosso" [Title/Abstract] OR "Mato Grosso do Sul" [Title/Abstract] OR Goiás [Title/Abstract] OR "Distrito Federal" [Title/Abstract] OR "Santa Catarina" [Title/Abstract] OR Paraná [Title/Abstract] OR "Rio Grande do Sul" [Title/Abstract])

**Figure S1:** PRISMA flowchart.

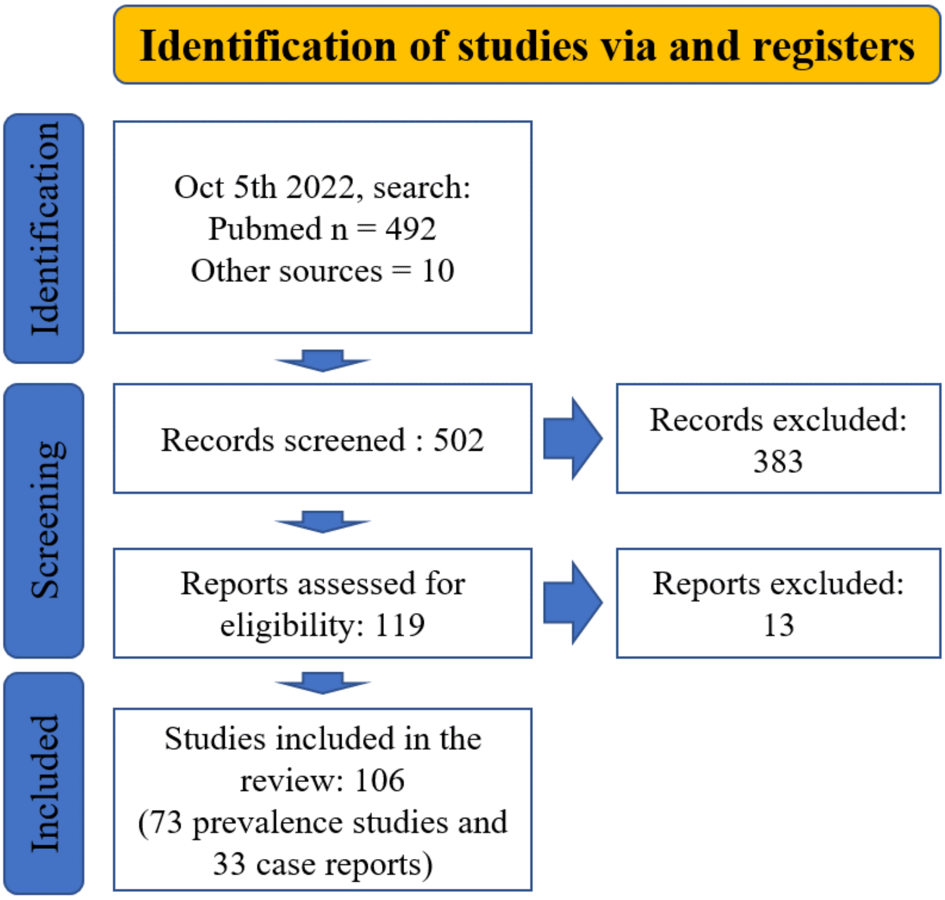

**Figure S2**: Quality assessment using the JBI Critical Appraisal Tools in Case Reports (A) and Prevalence Studies (B). Case Reports were evaluated according to the JBI Critical Appraisal Checklist for Case Reports, a list of 8 questions addressing the description of the: (1) patients' demography, (2) patients' history, (3) patients' symptomatology, (4) diagnostic methods, (5) intervention, (6) post-intervention clinical condition, (7) adverse or unanticipated events and (8) takeaway lessons. Prevalence Studies were evaluated according to the JBI Critical Appraisal Checklist for Studies Reporting Prevalence Data, a list of 9 questions addressing the: (1) sample frame, (2) sampling methods, (3) sample size, (4) description of the subjects, (5) coverage of the genomes, (6) validity of the variant calling method, (7) homogeneity of the variant calling process, (8) statistical analysis and (9) response rate. Questions #5, #6 and #7 of this instrument were not applicable for case reports once they are intended to address the intervention, which is not a part of the design of Genomic Surveillance Studies.

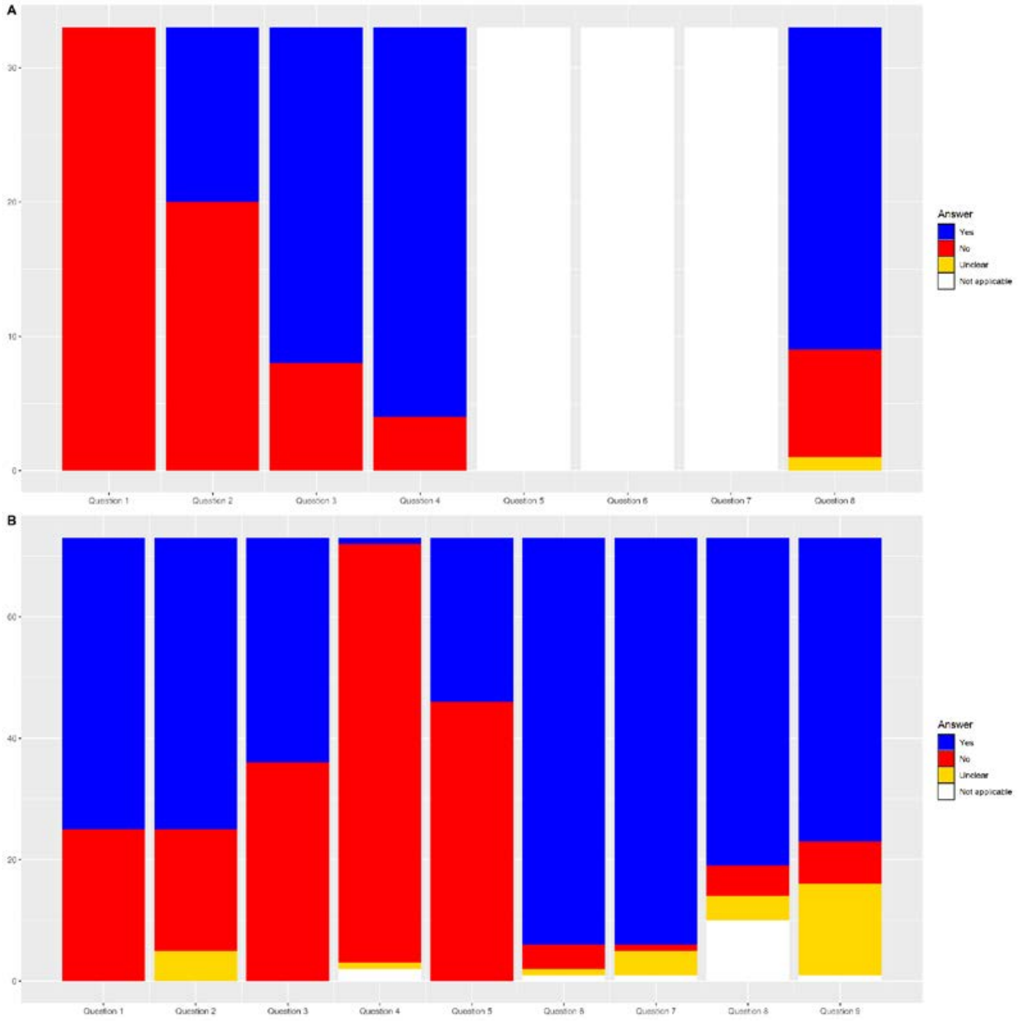

**Figure S3:** Sequencing metrics of the studies included in this review. The density histogram of the sample size (A), the absolute distribution and cumulative probabilities for the minimum (B) and medium (C) coverage as well as the minimum (D) and medium (E) genome span are shown in the panel.

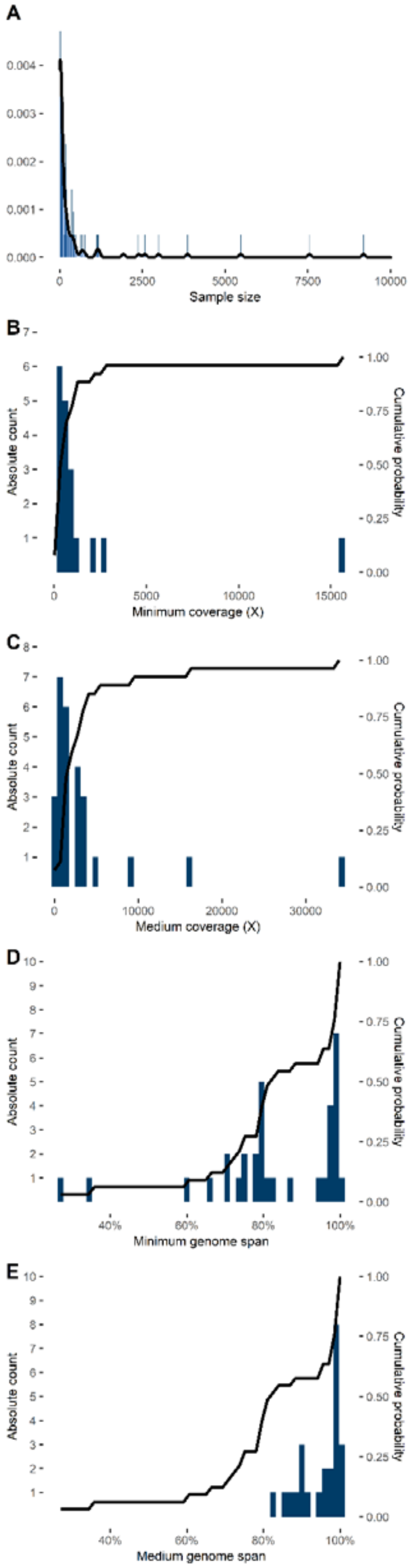

**Figure S4:** Keyword co-occurrence map. Five clusters with 806 links were found indicated by different colours. All keywords were used as analysis units. Keywords with at least three occurrences were included in the map (n = 54 out of 305). Links indicated keyword co-occurrence with the circle size weighted by the number of occurrences.

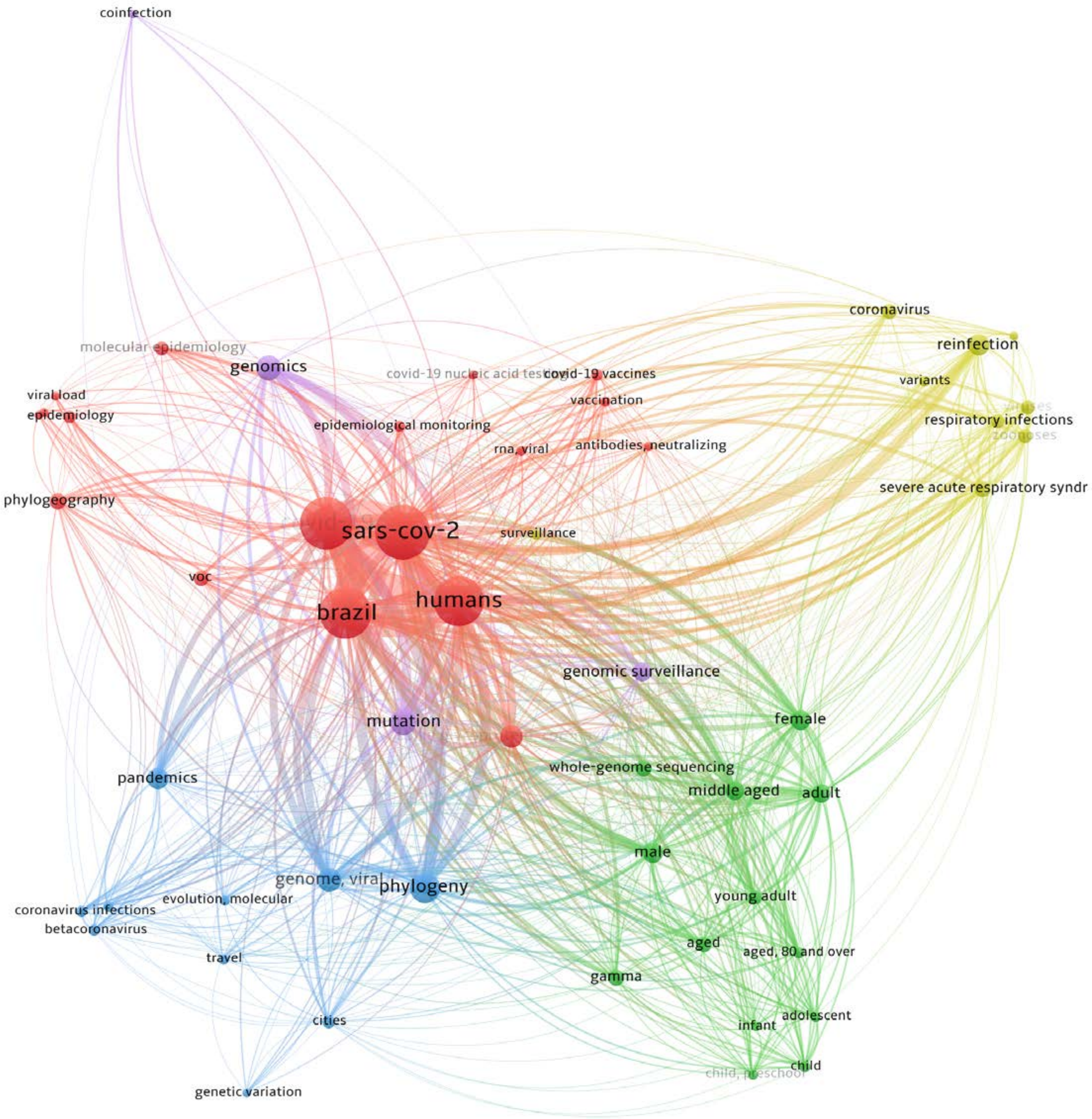

**Figure S5**: Journal citation map. Ten clusters with 172 links were found indicated by different colours. All journals were used as analysis units. Forty one of the 47 journals cited at least one other journal in the network and were included. Links indicated citation between journals with the circle size weighted by the number of published articles.

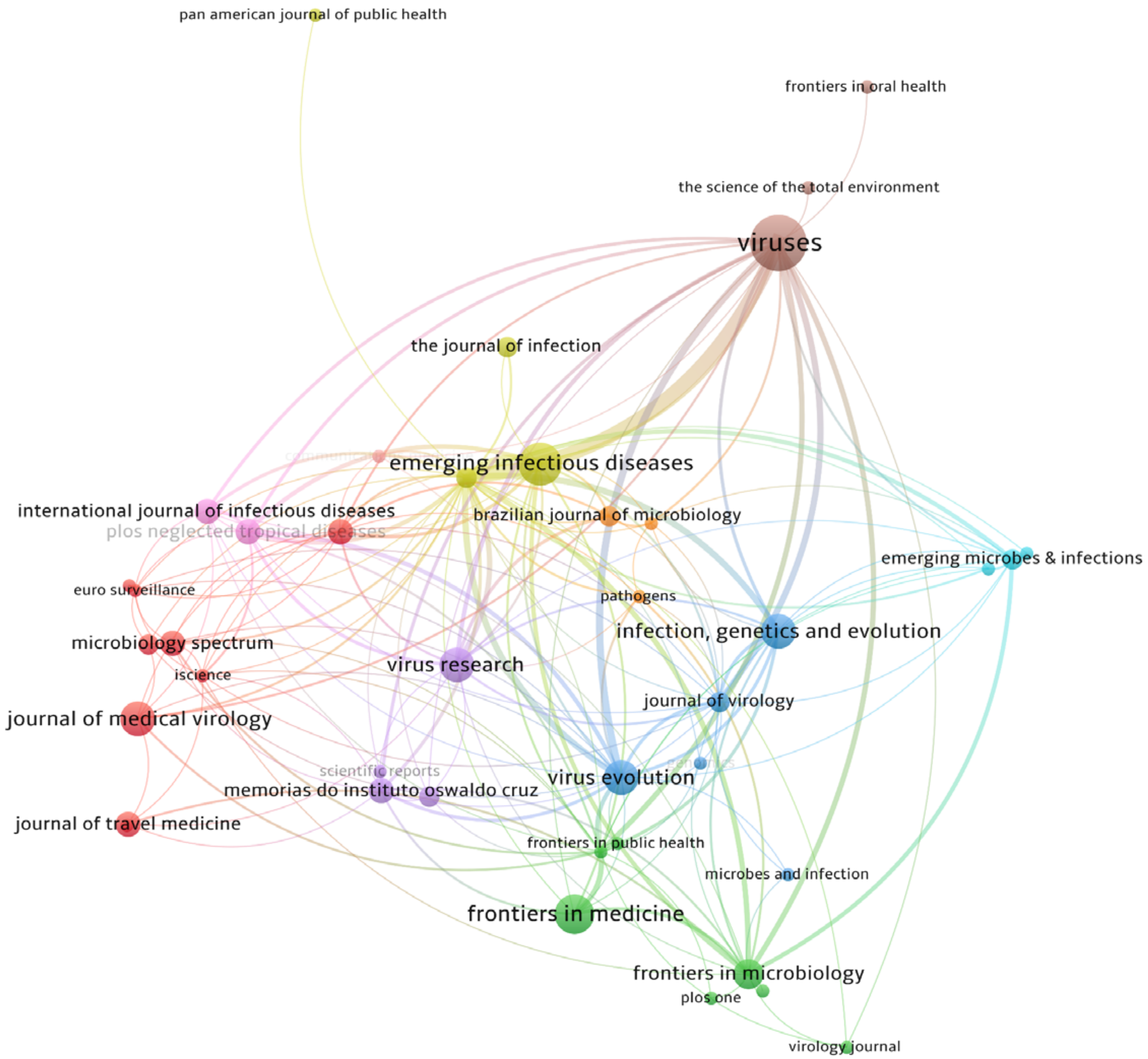

Supplement: Supplementary file 1 [file viruses-14-02715-s001.zip › SupplementaryMaterial_S1.pdf]
